# Vibration Characteristics Analysis of Moderately Thick Laminated Composite Plates with Arbitrary Boundary Conditions

**DOI:** 10.3390/ma12172829

**Published:** 2019-09-03

**Authors:** Zechang Xue, Qiuhong Li, Wenhao Huang, Yongxin Guo, Jiufa Wang

**Affiliations:** 1 College of Shipbuilding Engineering, Harbin Engineering University, Harbin 150001, China; 2 College of Mechanical and Electrical Engineering, Harbin Engineering University, Harbin 150001, China; 3 College of Automation, Harbin Engineering University, Harbin 150001, China; 4 No. 710 R&D Institute, CSIC, Yichang 443003, China

**Keywords:** moderately thick laminated composite plates, Mindlin theory, improved Fourier series, power flow, arbitrary elastic boundary, Hamilton principle

## Abstract

In this study, an improved Fourier series method is presented for the vibration modeling and analysis of moderately thick laminated composite plates with arbitrary boundary conditions, in which the vibration displacements are sought as the linear combination of a double Fourier cosine series and auxiliary series functions. The vibration model was established using the Hamilton energy principle. To study the vibration characteristics of laminated composite plates more comprehensively, firstly, the accuracy of the current results were validated via comparison with previous results and finite element method data. A parametric study was conducted on the effects of several key parameters, such as the h/b ratio, orientation and number of layers. In this section, both solutions are applicable to various combinations of boundary constraints, including classical boundary conditions and elastic-supported boundary conditions. Secondly, in order to identify the action position of vibration and the transmission of vibration energy, the response analysis of laminated plates was studied, and the power flow field for laminated plates was analyzed. Finally, a modal test was introduced to further verify the accuracy of the method in this paper.

## 1. Introduction

It is generally known that laminated composite plates, as basic structural components, are commonly applied in mechanical, aerospace, astronautic and civil engineering, among other fields. This is mainly because they are superior to conventional materials when high strength-to-weight and stiffness-to-weight ratios are required. Therefore, it is attractive for engineers and designers to research the material characteristics of the laminated composite plates.

At present, there are various theories of laminated plates, distinguished according to the differences in the displacement equation; these theories can be classified as follows: classical lamination theory (CLD) [1], first-order shear deformation theory (FSDT) [2,3], higher-order shear deformation theories (HSDT) [4,5,6], layer-wise lamination theory [7,8,9,10] and the three-dimensional elastic theory [11,12,13]. After establishing the vibration equation of composite laminates, there have been many studies on the vibration and response analysis of laminated composite plates. 

According to Yu et al. [14], the vibration characteristics of anisotropic rectangular plates under the mixed boundary conditions of solid support and simple support are analyzed based on the superposition principle. Ge et al. [15] studied the dynamic response of symmetrically orthogonal composite laminates on elastic foundations under in-plane preloading and transverse impact loads based on the modal superposition method. In this paper, the equation of motion for displacement description is deduced by using the higher-order shear deformation theory. Huang et al. [16] studied the free vibration characteristics of orthotropic rectangular plates subjected to internal compression on a two-parameter elastic foundation by the method of separated variables. Kshirsagar et al. [17] analyzed the free vibration and buckling of rectangular plates under arbitrary classical boundary conditions based on the superposition principle of infinite series with infinite truncation. Liu et al. [18] presented the exact closed solution of a rectangular plate by using the method of separating variables. The boundary conditions of plate structure in this paper are simply supported on one side, and have arbitrary classical boundary conditions on the other. Chung et al. [19] studied the vibration characteristics of rectangular plates under elastic boundary conditions by the Rayleigh‒Ritz method, where the Timoshenko beam function is used as the permissible deflection function. Liew et al. [20,21] presented the vibration characteristics of anisotropic plates and symmetrical composite laminates under mixed boundary conditions based on the subdomain and orthogonal polynomial methods. On this basis, they use the Reissner‒Mindlin theory and p-Ritz method to analyze the vibration characteristics of composite laminates and calculate the vibration frequencies of composite laminates under different boundary conditions, length-width ratios and width-thickness ratios. Cheung et al. [22] studied the free vibration characteristics of symmetrically laminated plates under point-supported boundary conditions by using a new displacement admissible function and the Rayleigh‒Ritz method. The allowable function is composed of a static beam function, which is different from the existing admissible function. The functions they set can satisfy both geometric boundary conditions and point-supported boundary conditions. Matsunaga et al. [23] analyzed the natural frequency and buckling stress of laminates by considering the influence of shear deformation thickness variation and moment of inertia, and expanded the vibration displacement function in power series. Mbakogu et al. [24] studied the bending problem of orthotropic rectangular plates under uniformly distributed loads by the Galerkin method. Dalaei et al. [25] solved the vibration problem of cantilever plates with anisotropic materials for the first time by using the extended Kantorovich method; they also obtained a closed-form high-precision solution in their paper. Bercin et al. [26] studied the bending and vibration of fully clamped plates by the Kantorovich method. Chen et al. [27,28,29,30] established a series of composite laminate models and studied the vibration, stability and large deformation of composite laminate plates. Luccioni et al. [31] presented the free vibration characteristics and stability of composite laminates by the finite element method, combining the classical laminate theory with the first-order shear deformation theory. Rao et al. [32] presented several finite element models to study the static, stability, impact and nonlinearity of laminated plates and shells. Shafiee et al. [33] analyzed the vibration characteristics of composite coupled plates by the finite element method. Huang et al. [34] studied the free vibration characteristics of rectangular plates with variable thickness based on the discrete method, and the characteristic equation of free vibration was obtained by the Green function. Liew et al. [35] presented the free vibration of symmetrical laminated plates by the moving least squares differential integral method. Song et al. [36,37,38] studied the free vibration of laminated plates by the local Radial Point Interpolation method; however, it is difficult to deal with the displacement boundary using a meshless method. Zhang et al. [39,40] presented an improved Fourier series method for the free vibration analysis of a moderately thick laminated composite rectangular plate with non-uniform boundary conditions. They also established a unified analysis model for vibration characteristics of composite laminated annular sector plates, circular sector plates, annular plates and circular plates with various elastic boundary conditions. Qin et al. [41] studied the free vibration analysis of composite laminated plates based on the Jacobi‒Ritz method.

Romanelli et al. [42] presented the dynamic response of a simply supported rectangular composite plate under local pressure based on the series method. Shen et al. [43,44] discussed the dynamic response of a cantilever plate on a Pastemak elastic foundation under temperature and transverse dynamic loads by the Rayleigh‒Ritz method, considering the influence of first-order shear deformation, foundation stiffness coefficient and temperature change. Khan et al. [45] presented the dynamic response of a composite cantilever plate under a uniform load by the variational method. Niyogi et al. [46] analyzed the free and forced vibration responses of composite laminates based on the nine-node element method, considering the influence of first-order transverse shear deformation and moment of inertia. Biswas et al. [47] studied the transient dynamic response behavior of multi-layered hybrid composite plates by a modified higher-order refined zigzag theory (HRZT). Zhang et al. [48] presented the free and forced vibration behaviors of thin, three-dimensionally coupled plate structures based on the dynamic stiffness method (DSM). Nefske et al. [49] presented a power flow finite element method, which is applied to the calculation of beam structures. In subsequent studies, power flow finite element method has been developed and gradually applied to the power flow analysis of coupled structures. Hambric et al. [50] calculated the structural sound intensity of a cantilever plate with stiffened members by the finite element method, and the bending, torsion and axial power flow were obtained. Li et al. [51] obtained the structural sound intensity vectors in three cases by finite element harmonic response analysis through calculating the surface admittance of a thin plate by the structural sound intensity method. Cieslik et al. [52] presented a method to introduce the bending moment and external force into the theoretical expression of structural vibration and sound intensity by using the complex mode theory. The vibration power flow of simply supported stiffened plates is calculated by the finite element method, and the energy flow distribution of stiffened plates is analyzed in this paper. Xing et al. [53] studied the power flow of structures under fluid‒structure interaction based on the substructure method. The energy flow density vector was used to represent the energy flow transmission path between substructures, and the energy flow transmission was visually shown by graphics. Wang et al. [54] studied the structural sound intensity characteristics of composite laminates under dynamic concentrated forces. An example of structural sound intensity was analyzed by using finite element software; in this paper, the results show that orthotropic laminates have different characteristics from isotropic laminates, and that the structural sound intensity characteristics of orthotropic laminates are influenced by the boundary conditions, number of layers and stacking sequence. Zhang et al. [55,56,57,58,59] presented the free and forced vibration behaviors of the laminated plate-cavity coupling system by means of the improved Fourier series method.

There have been many studies on thin plates with arbitrary boundary conditions. However, these studies have limitations for moderately thick composite plates with special supported boundary conditions, for instance, an arbitrary elastic boundary. In addition, because the Mindlin theory considers the shear deformation along the thickness direction compared with the equivalent single-layer theory used in thin plates when studying the vibration of plates, the results obtained by the Mindlin theory are more accurate. On the other hand, most of the recent research merely discusses the vibration analysis, while the research on energy transfer is insufficient. Nevertheless, in many engineering applications, such as in the design of vehicles, it is necessary to comprehend the energy transfer path in order to reduce noise and vibration. Yan [60] points out that, compared with the traditional path analysis method, power flow analysis not only describes the speed of energy transfer or transformation, but also can be used to characterize the flow of vibration energy in the system. An et al. [61] studied the transmission path in hydraulic pipelines by using vibration power flow. Inoue et al. [62,63,64] studied the relationship between the structural noise response and the total power flow transmitted to the receiving structure, and they presented that there is a certain similarity between the response spectrum of structural noise and the total power spectrum transmitted to the receiving structure in a wide frequency range. Lee et al. [65,66] used the method of measuring vibration power flow to analyze the transmission paths of occupant interior structural noise caused by vehicle transmission system; they identified the main transmission paths according to the power flow, and modified the body structure with modal analysis to reduce the structural noise. Wang [67] studied the transmission characteristic of energy flows of micro-vibrations in spacecraft structures. Therefore, the study of power flow analysis of structural component is essential. 

In this paper, a mechanics model is presented, based on the Mindlin theory, in order to research its vibration characteristics with arbitrary boundary conditions. Three kinds of restraining springs (translational, rotational and torsional), attached to each edge, are introduced to establish the general structure model of laminated composite plates. In addition, a modified Fourier series method is introduced to describe the vibration displacement function of the structure to study the influences of the structural style and the boundary conditions on the vibration characteristics of laminated composite plates. Moreover, the vibration model of the laminated composite plates with arbitrary boundary conditions is established, which is based on the Hamilton energy variation principle. This paper also focuses on the effect on vibration frequency while changing various key parameters (such as the ratio between thickness and width, the number of layers, as well as the laying angle between two layers). In the following section, several numerical examples of the free vibration of a laminated composite plate with arbitrary boundary conditions are presented, which can serve as references for future engineering. The current results were checked against previous results and achieved good agreement. Moreover, to analyze the vibration characteristics of laminated composite plates more comprehensively, the present work discusses the vibration response characteristic of the laminate, in terms of energy, through harmonic response and power flow analysis. In this section, we discuss the periodic response of a continuous periodic load in a structural system; in addition, we found the distribution character of energy transfer through changing the position of actions and boundary conditions. Finally, we introduce a test to further verify the accuracy of the presented method, in which the experimental data coincide with the theoretical value. 

## 2. Theoretical Formulations

**Figure 1:**
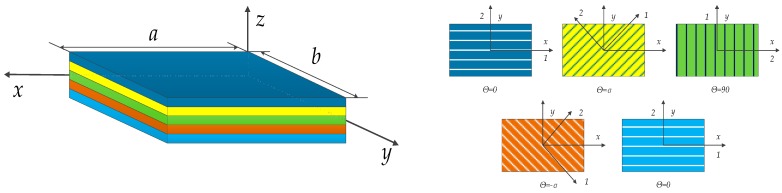
Abbreviated drawing of laminated composite plates.

The mechanical properties of the composite laminate structure were related to the mechanical properties and thickness of the single-layer board, as well as the fiber laying direction and sequence and the number of layers. The laminated plate shown in Figure 1 consisted of five layers. The angle of the first layer was 0°, and the other four layers were expressed as *α*, 90°, −*α* and 0°, respectively. The principal axis coordinate system of each layer in laminated composite plates was O_12_, and the global coordinate system was O_xy_. The ply-angle of the fiber can be expressed by *θ*, and the positive direction was identified when the *x*-axis rolled towards the 1 axis in an anticlockwise direction. The material of the plate structure in this chapter was a fiber-reinforced composite material, which was composed of matrix and fiber. The matrix was mainly used to support and protect the fiber, while the fiber was enhanced and mainly supported. In addition, the coupling vibration of bending and stretching of composite laminate plates is not considered in this chapter.

For the plate model based on the assumptions of the first-order shear deformation theory, the deformation of the plate was continuous. Thus, on the basis of not considering the in-plane vibration of plates, the displacement field can be expressed as

(1) $$u(x,y,z)=z\psi_{x}(x,y),$$

(2) $$v(x,y,z)=z\psi_{y}(x,y),$$

(3) w(x,y,z)=w(x,y)

where *u*, *v* and *w* are the displacement in the *x*-, *y*- and *z*- directions, respectively, and *ψ*_x_ and *ψ*_y_ are the rotation in the *x*- and *y*- directions, respectively. Therefore, the stress‒strain relationship in coordinates, based on the small deflection theory, can be written as follows:

(4) $$\left\{\begin{array}{c} \varepsilon_{x}\\ \varepsilon_{y}\\ \gamma_{xy}\\ \gamma_{yz}\\ \gamma_{xz} \end{array}\right\}=\;\left\{z\begin{array}{c} z\partial \psi_{x}/\partial x\\ z\partial \psi_{y}/\partial y\\ (\partial \psi_{x}/\partial x+\partial \psi_{y}/\partial y)\\ \psi_{x}+\partial w/\partial x\\ \psi_{y}+\partial w/\partial y \end{array}\right\}.$$

The corresponding stresses are obtained in terms of the generalized Hooke’s law:

(5) $$\left\{\begin{array}{l} \sigma_{x}\\ \sigma_{y}\\ \tau_{xy} \end{array}\right\}=\left[\begin{array}{ccc} \bar{Q_{11}^{k}} & \bar{Q_{12}^{k}} & \bar{Q_{16}^{k}}\\ \bar{Q_{12}^{k}} & \bar{Q_{22}^{k}} & \bar{Q_{26}^{k}}\\ \bar{Q_{16}^{k}} & \bar{Q_{26}^{k}} & \bar{Q_{66}^{k}} \end{array}\right]\left\{\begin{array}{l} \varepsilon_{x}\\ \varepsilon_{y}\\ \gamma_{xy} \end{array}\right\},\left\{\begin{array}{l} \tau_{\mathrm{yz}}\\ \tau_{\mathrm{xz}} \end{array}\right\}=\left[\begin{array}{cc} \bar{Q_{44}^{k}} & \bar{Q_{45}^{k}}\\ \bar{Q_{45}^{k}} & \bar{Q_{55}^{k}} \end{array}\right]\left\{\begin{array}{l} \gamma_{yz}\\ \gamma_{xz} \end{array}\right\},$$

where the normal stresses are $\sigma_{x}$ and $\sigma_{y}$ in the *x* and *y* directions, respectively, and the shear stresses are $\tau_{\mathrm{yz}}$, $\tau_{\mathrm{xz}}$ and $\tau_{xy}$ in the *x*, *y* and *z* coordinate system, respectively. The lamina stiffness coefficients are $\bar{Q_{ij}^{k}}$(*i*, *j* = 1, 2, 4, 5, 6), which can be expressed as follows:

(6) $$\begin{array}{l} \bar{Q_{11}^{k}}=Q_{11}^{k}m^{4}+2(Q_{12}^{k}+2Q_{66}^{k})m^{2}n^{2}+Q_{22}^{k}n^{4}\\ \bar{Q_{12}^{k}}=(Q_{11}^{k}+Q_{22}^{k}+4Q_{66}^{k})m^{2}n^{2}+Q_{12}^{k}(m^{4}+n^{4})\\ \bar{Q_{22}^{k}}=Q_{11}^{k}n^{4}+2(Q_{12}^{k}+2Q_{66}^{k})m^{2}n^{2}+Q_{22}^{k}m^{4}\\ \bar{Q_{16}^{k}}=(Q_{11}^{k}-Q_{12}^{k}-2Q_{66}^{k})m^{3}n+(Q_{12}^{k}-Q_{22}^{k}+2Q_{66}^{k})mn^{3}\\ \bar{Q_{26}^{k}}=(Q_{11}^{k}-Q_{12}^{k}-2Q_{66}^{k})mn^{3}+(Q_{12}^{k}-Q_{22}^{k}+2Q_{66}^{k})m^{3}n\\ \bar{Q_{66}^{k}}=(Q_{11}^{k}+Q_{22}^{k}-2Q_{12}^{k}-2Q_{66}^{k})m^{2}n^{2}+Q_{66}^{k}(m^{4}+n^{4})\\ \bar{Q_{44}^{k}}=Q_{44}^{k}m^{2}+Q_{55}^{k}n^{2}\\ \bar{Q_{45}^{k}}=(Q_{55}^{k}-Q_{44}^{k})mn\\ \bar{Q_{55}^{k}}=Q_{55}^{k}m^{2}+Q_{44}^{k}n^{2} \end{array},$$

where $m=\cos\theta^{k}$,$n=\sin\theta^{k}$, in which the included angle is simply represented as *θ* between the principal direction of the layer and the *x*–axis. The lamina elastic coefficients $Q_{ij}^{k}$ in Equation (6) can be obtained from the material properties of the *k*th orthotropic lamina layer:

(7) $$\begin{array}{l} Q_{11}^{k}=\frac{E_{1}}{1-\mu_{12}\mu_{21}},\;Q_{12}^{k}=\frac{\mu_{12}E_{2}}{1-\mu_{12}\mu_{21}},\;Q_{12}^{k}=\frac{E_{2}}{1-\mu_{12}\mu_{21}}\\ Q_{44}^{k}=G_{23},\;Q_{55}^{k}=G_{13},\;Q_{66}^{k}=G_{12}, \end{array}$$

where $E_{1}$ and $E_{2}$ denote the longitudinal modulus and the transverse modulus, $\mu_{12}$ is the major Poisson’s ratio and the other Poisson’s ratio $\mu_{21}$ can be obtained by $\mu_{12}E_{2}=\mu_{21}E_{1}$. $G_{12}$, $G_{13}$ and $G_{23}$ are the shear moduli.

Through taking the unit body of unit length from the plate in the *x*/*y* direction, we can synthesize the stress component on the cross section of the unit body into an internal force per unit width. Therefore, the bending moment $M_{x}$ and $M_{y}$, the torque $M_{xy}$ and $M_{yx}$ and the shear force $Q_{x}$ and $Q_{y}$ can be expressed as

(8) $$\begin{array}{l} M_{x}=\int_{-h/2}^{h/2}\sigma_{x}zdz=\sum_{k=1}^{n}\int_{z_{k}}^{z_{k+1}}\sigma_{x}^{k}zdz,\\ M_{y}=\int_{-h/2}^{h/2}\sigma_{y}zdz=\sum_{k=1}^{n}\int_{z_{k}}^{z_{k+1}}\sigma_{y}^{k}zdz, \end{array}$$

(9) $$M_{xy}=M_{yx}=\int_{-h/2}^{h/2}\tau_{xy}zdz=\sum_{k=1}^{n}\int_{z_{k}}^{z_{k+1}}\tau_{xy}^{k}zdz,$$

(10) $$\begin{array}{l} Q_{x}=\kappa \int_{-h/2}^{h/2}\tau_{xz}dz=\kappa \sum_{k=1}^{n}\int_{z_{k}}^{z_{k+1}}\tau_{xz}^{k}dz,\\ Q_{y}=\kappa \int_{-h/2}^{h/2}\tau_{yz}dz=\kappa \sum_{k=1}^{n}\int_{z_{k}}^{z_{k+1}}\tau_{yz}^{k}dz, \end{array}$$

where κ is the shear correction factor, and $\sigma_{x}^{k}$, $\sigma_{y}^{k}$, $\tau_{xy}^{k}$, $\tau_{xz}^{k}$ and $\tau_{yz}^{k}$ are the corresponding stresses of the *k*th layer.

**Figure 2:**
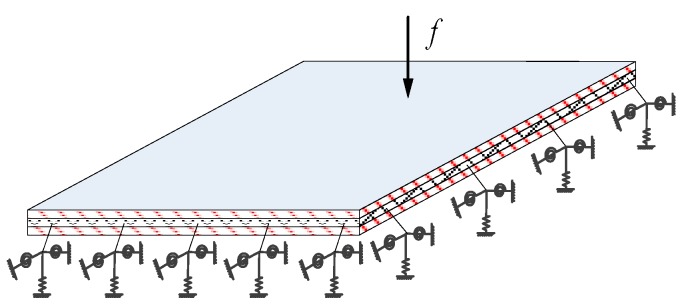
A laminated composite plate with arbitrary elastic boundary support.

Consider a structural model of laminated composite plates that is elastically restrained along its four edges, as shown in Figure 2. The laminated composite plates may also be loaded with three kinds of restraining springs (translational, rotational and torsional) at arbitrary locations, and the boundary conditions can be easily obtained by changing the stiffness of the springs.

According to Hamilton’s principle, the vibration equation can be easily established. For a holonomic mechanical system with n generalized degrees of freedom, the generalized coordinates are *q*_s_ (s = 1, 2, … n), the Lagrange function is $L(q_{s},{\dot{q}}_{s},t)=T-U-W$, where *T*, *U* and *W* are the kinetic energy, potential energy and the external work, respectively. The Hamilton action is defined as follows:

(11) $$S=\int_{t_{o}}^{t_{1}}L(q_{s},{\dot{q}}_{s},t)dt.$$

For the laminated composite plate in Figure 2, the Hamilton equation of the plate can be expressed as follows:

(12) $$\delta \int_{t_{0}}^{t_{1}}\left(V-T-W_{\mathrm{ext}}\right)dt=0,$$

where *V*, *T* and *W_ext_*, denote the total potential, the total kinetic energy and the external potential energy, respectively. The total potential includes the bending potential energy of the plate and the elastic potential energy of springs. The total potential energy can be written as follows:

(13) $$V=V_{\mathrm{plate}}+V_{\mathrm{spring}},$$

where *V*_spring_ is the elastic potential energy of springs, *V*_plate_ is the bending potential energy of the plate and these terms can be explicitly expressed as follows.

The elastic potential energy of springs on the four edges can be written as follows:

(14) $$\begin{array}{ll} V_{spring} & =\frac{1}{2}\int_{0}^{b}\left\{\begin{array}{l} {\left[k_{x0}w^{2}+K_{x0}\psi_{x}^{2}+K_{yx0}\psi_{y}^{2}\right]}_{x=0}\\ +{\left[\left[k_{xa}w^{2}+K_{xa}\psi_{x}^{2}+K_{yxa}\psi_{y}^{2}\right]\right]}_{x=a} \end{array}\right\}dy\\ & \;+\frac{1}{2}\int_{0}^{a}\left\{\begin{array}{l} {\left[k_{y0}w^{2}+K_{y0}\psi_{y}^{2}+K_{xy0}\psi_{x}^{2}\right]}_{y=0}\\ +{\left[k_{yb}w^{2}+K_{yb}\psi_{y}^{2}+K_{xyb}\psi_{x}^{2}\right]}_{y=b} \end{array}\right\}dx \end{array}.$$

The stress relationship can be evaluated according to the stress‒strain relationship. On the basis of the elastic mechanic theory, the relationship between elastic potential energy and stress‒strain is expressed as follows:

(15) $$V_{plate}=\frac{1}{2}\int_{V}\varepsilon^{Τ}\sigma dV=\frac{1}{2}\int_{S}\varepsilon_{1}^{T}\Theta \varepsilon_{1}dS.$$

In this equation, ***Θ*** is given by

(16) $$\Theta =\left[\begin{array}{ccccc} D_{11} & D_{12} & D_{16} & 0 & 0\\ D_{21} & D_{22} & D_{26} & 0 & 0\\ D_{16} & D_{26} & D_{66} & 0 & 0\\ 0 & 0 & 0 & A_{55} & A_{45}\\ 0 & 0 & 0 & A_{45} & A_{44} \end{array}\right],$$

and ***ε***_1_ is given by

(17) $$\varepsilon_{1}=\left\{\begin{array}{c} \partial \psi_{x}/\partial x\\ \partial \psi_{y}/\partial y\\ (\partial \psi_{x}/\partial x+\partial \psi_{y}/\partial y)\\ \psi_{x}+\partial w/\partial x\\ \psi_{y}+\partial w/\partial y \end{array}\right\}.$$

The stiffness can be defined as

(18) $$D_{ij}=\frac{1}{3}\sum_{q=1}^{n}Q_{ij}^{\left(q\right)}\left(h_{k}^{3}-h_{k-1}^{3}\right)\;i,j\;=\;1,2,6,$$

(19) $$A_{ij}=\kappa \sum_{q=1}^{n}Q_{ij}^{\left(q\right)}\left(h_{k}-h_{k-1}\right)\;i,j\;=\;4,5.$$

Therefore, Equation (5) can be expressed as follows:

(20) $$\begin{array}{ll} V_{plate} & =\frac{1}{2}\int_{0}^{a}\int_{0}^{b}\{D_{11}{\left(\frac{\partial \psi_{x}}{\partial x}\right)}^{2}+D_{22}{\left(\frac{\partial \psi_{y}}{\partial y}\right)}^{2}+2D_{12}\left(\frac{\partial \psi_{x}}{\partial x}\frac{\partial \psi_{y}}{\partial y}\right)\\ & \;+2D_{16}\frac{\partial \psi_{x}}{\partial x}\left(\frac{\partial \psi_{x}}{\partial y}+\frac{\partial \psi_{y}}{\partial x}\right)\;+2D_{26}\frac{\partial \psi_{y}}{\partial y}\left(\frac{\partial \psi_{x}}{\partial y}+\frac{\partial \psi_{y}}{\partial x}\right)\;+D_{66}{\left(\frac{\partial \psi_{x}}{\partial y}+\frac{\partial \psi_{y}}{\partial x}\right)}^{2}\\ & \;+A_{44}{\left(\frac{\partial w}{\partial y}+\psi_{y}\right)}^{2}+2A_{45}\left(\frac{\partial w}{\partial y}+\psi_{y}\right)\left(\frac{\partial w}{\partial x}+\psi_{x}\right)+A_{55}{\left(\frac{\partial w}{\partial x}+\psi_{x}\right)}^{2}\}dxdy \end{array}.$$

The total kinetic energy of the laminated composite plate is given by

(21) $$T=\frac{1}{2}\rho h\omega^{2}\int_{0}^{a}\int_{0}^{b}\left\{w^{2}+\frac{1}{12}h^{2}\left(\psi_{x}^{2}+\psi_{y}^{2}\right)\right\}dxdy.$$

The external potential energy of the laminated composite plate can be expressed as

(22) $$W_{\mathrm{ext}}=\int_{0}^{a}\int_{0}^{b}f(x,y)w(x,y)dxdy,$$

where *w* is the flexural displacement; *a* and *b* are the plate dimension in *x* direction and the plate dimension in *y* direction, respectively; *ρ* and *h* are the mass density and the thickness of the plate, respectively, and *f* (*x*, *y*) = *F δ* (*x* − *x*_0_) (y − *y*_0_), where *δ* is the delta function, *F* is the amplitude of the stresses and *x*_0_ and *y*_0_ are the position coordinates of force. *k*_x0_ and *k*_xa_ (*k*_y0_ and *k*_yb_) are the transverse spring constants, *K*_x0_ and *K*_xa_ (*K*_y0_ and *K*_yb_) are the rotational spring constants and *K*_yx0_ and *K*_yxa_ (*K*_xy0_ and *K*_xyb_) are the torsional spring constants at *x* = 0 and *x* = a (*y* = 0 and *y* = b), respectively.

The displacement function is expanded as a single Fourier series plus an auxiliary polynomial function. The auxiliary function is used to overcome the discontinuities of the resilient boundary, the transverse displacement function *w*(*x*, *y*).

(23) $$w(x,y)=\sum_{m=0}^{\infty }\sum_{n=0}^{\infty }A_{mn}\cos(\lambda_{m}x)\cos(\lambda_{n}y)+p(x,y),$$

where *p*(*x*,*y*) can be expressed as

(24) $$\begin{array}{ll} p(x,y)= & p_{1}(x,y)\sum_{m=0}^{\infty }d_{1m}^{1}\cos(\lambda_{m}x)+p_{2}(x,y)\sum_{m=0}^{\infty }d_{2m}^{1}\cos(\lambda_{m}x)\\ & +p_{3}(x,y)\sum_{n=0}^{\infty }f_{1n}\cos(\lambda_{n}y)+p_{4}(x,y)\sum_{n=0}^{\infty }f_{2n}\cos(\lambda_{n}y) \end{array},$$

where the auxiliary polynomial based on trigonometric function can be written as follows:

(25) $$p_{1}(x,y)=\xi_{1b}(y)=\frac{b}{2\pi }\sin\frac{\pi y}{2b}+\frac{b}{2\pi }\sin\frac{3\pi y}{2b},$$

(26) $$p_{2}(x,y)=\xi_{2b}(y)=-\frac{b}{2\pi }\cos\frac{\pi y}{2b}+\frac{b}{2\pi }\cos\frac{3\pi y}{2b},$$

(27) $$p_{3}(x,y)=\xi_{1a}(x)=\frac{a}{2\pi }\sin\frac{\pi x}{2a}+\frac{a}{2\pi }\sin\frac{3\pi x}{2a},$$

(28) $$p_{4}(x,y)=\xi_{2a}(x)=-\frac{a}{2\pi }\cos\frac{\pi x}{2a}+\frac{a}{2\pi }\cos\frac{3\pi x}{2a},$$

where *λ*_m_ = *m*π/*a*, *λ*_n_ = *n*π/*b*; *a* is length and *b* is width and *A*_mn_, $d_{1m}^{1}$,$d_{2m}^{1}$,$f_{1n}^{1}$ and $f_{2n}^{1}$ are the expansion coefficients. 

Therefore, function *w*(*x*, *y*) can be rewritten as follows:

(29) $$\begin{array}{ll} w(x,y)= & \sum_{m=0}^{\infty }\sum_{n=0}^{\infty }A_{mn}\cos(\lambda_{m}x)\cos(\lambda_{n}y)\\ & +\sum_{m=0}^{\infty }d_{1m}^{1}\xi_{1b}(y)\cos(\lambda_{m}x)+\sum_{m=0}^{\infty }d_{2m}^{1}\xi_{2b}(y)\cos(\lambda_{m}x)\\ & +\sum_{n=0}^{\infty }f_{1n}^{1}\xi_{1a}(x)\cos(\lambda_{n}y)+\sum_{n=0}^{\infty }f_{2n}^{1}\xi_{2a}(x)\cos(\lambda_{n}y) \end{array}.$$

Similarly, the rotational displacement function *Ψ*_x_(x, y) and *Ψ*_y_ (x, y) can be easily obtained as follows:

(30) $$\begin{array}{ll} \psi_{x}(x,y)= & \sum_{m=0}^{\infty }\sum_{n=0}^{\infty }B_{mn}\cos(\lambda_{m}x)\cos(\lambda_{n}y)\\ & +\sum_{m=0}^{\infty }d_{1m}^{2}\xi_{1b}(y)\cos(\lambda_{m}x)+\sum_{m=0}^{\infty }d_{2m}^{2}\xi_{2b}(y)\cos(\lambda_{m}x)\\ & +\sum_{n=0}^{\infty }f_{1n}^{2}\xi_{1a}(x)\cos(\lambda_{n}y)+\sum_{n=0}^{\infty }f_{2n}^{2}\xi_{2a}(x)\cos(\lambda_{n}y) \end{array},$$

(31) $$\begin{array}{ll} \psi_{y}(x,y)= & \sum_{m=0}^{\infty }\sum_{n=0}^{\infty }C_{mn}\cos(\lambda_{m}x)\cos(\lambda_{n}y)\\ & +\sum_{m=0}^{\infty }d_{1m}^{3}\xi_{1b}(y)\cos(\lambda_{m}x)+\sum_{m=0}^{\infty }d_{2m}^{3}\xi_{2b}(y)\cos(\lambda_{m}x)\\ & +\sum_{n=0}^{\infty }f_{1n}^{3}\xi_{1a}(x)\cos(\lambda_{n}y)+\sum_{n=0}^{\infty }f_{2n}^{3}\xi_{2a}(x)\cos(\lambda_{n}y) \end{array},$$

where *B_mn_*, $d_{1m}^{2}$,$d_{2m}^{2}$,$f_{1n}^{2}$,$f_{2n}^{2}$,*C*_mn_, $d_{1m}^{3}$,$d_{2m}^{3}$,$f_{1n}^{3}$ and $f_{2n}^{3}$ are the expansion coefficients.

Substitution of Equations (17)–(19) into Equation (11) leads to a series of equations, and these equations can be rewritten in matrix form as

(32) $$(K-\rho h\omega^{2}M)A=F,$$

where ***K*** is the stiffness matric, ***M*** is the mass matric, ***A*** is the unknown coefficient vector and ***F*** is the force vector. The detailed expressions of ***K*** and ***M*** are shown in the Appendix A.

(33) $$\begin{array}{l} A=[A_{00},A_{01},\cdot \cdot \cdot A_{MN},d_{10}^{1},d_{11}^{1},\cdot \cdot \cdot d_{2M}^{1},f_{10}^{1},f_{11}^{1},\cdot \cdot \cdot f_{2N}^{1},\\ \;B_{00},B_{01},\cdot \cdot \cdot B_{MN},d_{10}^{2},d_{11}^{2},\cdot \cdot \cdot d_{2M}^{2},f_{10}^{2},f_{11}^{2},\cdot \cdot \cdot f_{2N}^{2},\\ {\;C_{00},C_{01},\cdot \cdot \cdot C_{MN},d_{10}^{3},d_{11}^{3},\cdot \cdot \cdot d_{2M}^{3},f_{10}^{3},f_{11}^{3},\cdot \cdot \cdot f_{2N}^{3}]}^{T} \end{array},$$

(34) $$K=\left[\begin{array}{cccc} K_{1,1} & K_{1,2} & \cdots & K_{1,15}\\ K_{2,1} & K_{2,2} & \cdots & K_{2,15}\\ \vdots & \vdots & \ddots & \vdots \\ K_{15,1} & K_{15,2} & \cdots & K_{15,15} \end{array}\right],$$

(35) $$M=\left[\begin{array}{cccc} M_{1,1} & M_{1,2} & \cdots & M_{1,15}\\ M_{2,1} & M_{2,2} & \cdots & M_{2,15}\\ \vdots & \vdots & \ddots & \vdots \\ M_{15,1} & M_{15,2} & \cdots & M_{15,15} \end{array}\right].$$

In order to determine the modal characteristics of the laminated composite plate, one needs to solve the characteristic equation by setting the external force vector ***F*** in Equation (20) to zero. Obviously, the natural frequencies and eigenvectors of the laminated composite plate can now be easily obtained by solving a standard matrix Eigen problem. Since the components of each eigenvector are actually the expansion coefficients of the Fourier series, the corresponding mode shape can be directly determined by substituting the eigenvector in Equation (17) into Equation (19).

On the other hand, when the external force vector F is not zero, it can be used to study the response analysis of laminates. The response obtained is a harmonic response when the external excitation force is a simple harmonic force.

(36) $$A={\left(K-\rho h\omega^{2}M\right)}^{-1}F.$$

The lateral displacement field and the corner of the bending vibration of the plate structure at the excitation frequency by substituting the coefficient vector *A* in Equation (17) into Equation (19).

To solve the structural vibration and noise radiation problems, vibration power flow leads to a more comprehensive evaluation of the vibration response characteristics of the structure. It can intuitively identify the position of the vibration and the transmission path of the vibration energy, providing a better evaluation index and basis for the vibration control of the structure.

The power flow intensity *I*(*x*, *y*) at any point in the Mindlin plate structure can be expressed as follows:

(37) $$I(x,y)=\sqrt{|I_{x}(x,y){|}^{2}+|I_{y}(x,y){|}^{2}},$$

where *I_x_*(*x*,*y*) and *I_y_*(*x*,*y*) are the components of *I*(*x*, *y*) along the *x* and *y* axes, respectively.

(38) $$I_{x}=-\frac{1}{2}Re\left\{Q_{x}{\left(\frac{\partial w}{\partial t}\right)}^{*}-M_{x}{\left(\frac{\partial \psi_{x}}{\partial t}\right)}^{*}-M_{xy}{\left(\frac{\partial \psi_{y}}{\partial t}\right)}^{*}\right\},$$

(39) $$I_{y}=-\frac{1}{2}Re\left\{Q_{y}{\left(\frac{\partial w}{\partial t}\right)}^{*}-M_{y}{\left(\frac{\partial \psi_{y}}{\partial t}\right)}^{*}-M_{yx}{\left(\frac{\partial \psi_{x}}{\partial t}\right)}^{*}\right\}.$$

Therefore, the power flow intensity *I*(*x*, *y*) at any point can be easily obtained by substituting Equations (8)–(10) into Equation (38) and Equations (29)–(31) into Equation (39).

## 3. Results and Discussion

### 3.1. Modal Characteristics of Laminated Composite Plates

Consider a laminated composite plate with arbitrary elastic boundary support, with the dimensions of *a* × *b*, which is illustrated in Figure 2. If three kinds of restraining springs (translational, rotational and torsional) are assumed to be distributed uniformly along each edge, then all the classical boundary conditions, as well as their combinations, can be easily obtained by simply setting the spring coefficients to zero or infinity. For the sake of convenience, *C* denotes the clamped cases, *E* denotes the elastic bearing cases, *F* denotes the free-boundary condition and *S* represents the simply supported cases. Taking the edge of x = 0 as an example, the spring stiffness is shown in Table 1 when the boundary condition is *F*, *S*, *C* or *E*, respectively. (Note that the elastic boundary can be chosen arbitrarily, and the exact value of E can be transformable in different examples.) 

First, the relationship between the vibration solution of a rectangular plate based on the energy principle and the analytical solution based on the governing equation and the boundary condition equation was studied. The laminate was a single-layer board and the material was an isotropic material, the same as in [18].

In Table 2, the first five non-dimensional frequency parameters are shown (Ω = (*ωb*^2^/*π*^2^)(*ρh*/*D*)^1/2^). It can be seen from the table that the deviations of the results obtained by the present method and the analytical method were all within 0.022. The results obtained were almost identical, which verified that the energy method resulted in an exact solution when the permissible displacement function satisfied both the displacement boundary condition and the force boundary condition.

In the following calculations, the laminated composite was an orthogonal symmetrical laminate, which was composed of three layers of unit plates of equal thickness—that is, the layup direction of the unit plate was 0°/90°/0°. The physical parameters are specified as *E*_x_/*E*_y_ = 40, *G*_12_ = 3/5*E*_y_, *G*_23_ = 1/2*E*_y_, *G*_13_ = 3/5*E*_y_, *v*_y_ = 1/4, *v*_x_ = 0.00625, S_16_ = S_26_ = S_45_ = 0, Poisson’s ratio *μ* = 0.3, shear correction factor *k* = 5/6.

To verify the convergence of the solution, Table 3 compares the first seven non-dimensional modal frequency parameters of the laminated composite plate under different cutoff values. For illustrative purposes, one assumes that a *SSFF* laminated composite plate system shown in Table 3 is defined by the dataset: D_0_ = *Eh*^3^/(12(1 − *v*_x_*v*_y_)); *a*/*b* = 1; *h*/*b* = 0.2; with *E* representing the larger value between *E*_x_ and *E*_y_. At the same time, a comparison of the solution in [21] is also presented. It is seen that the maximum deviation of the first seven non-dimensional modal frequency parameters obtained in both cases (*M = N* = 4 and *M* = *N* = 16) was 1.4%; on the other hand, when the cutoff value was more than 10, the results were almost invariant. Thus, it was evident that just a few terms can lead to excellent prediction and the current solution shows remarkable convergence.

To further verify the accuracy of the method from a modal perspective, the first five non-dimensional frequency parameters are shown for the plates of various thickness–width ratios and boundary conditions in Table 4 and Table 5. Meanwhile, the results calculated by using the separate variable method in [21], the Rayleigh‒Ritz method in [18] and the results calculated by FEA are also presented in Table 4 and Table 5. It is seen that the present method has led to excellent agreement with the classical solution. 

**Figure 3:**
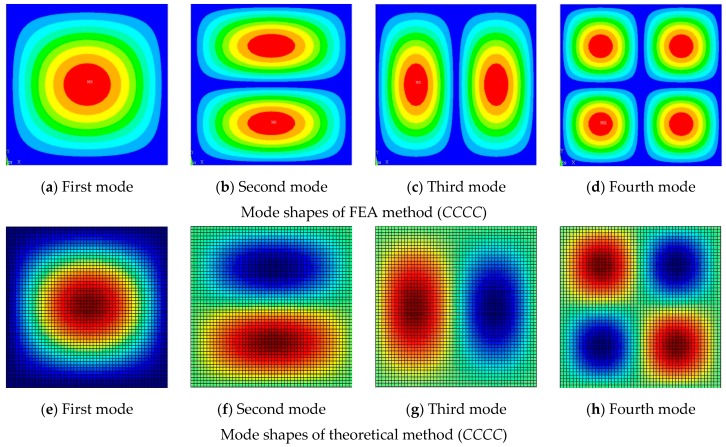

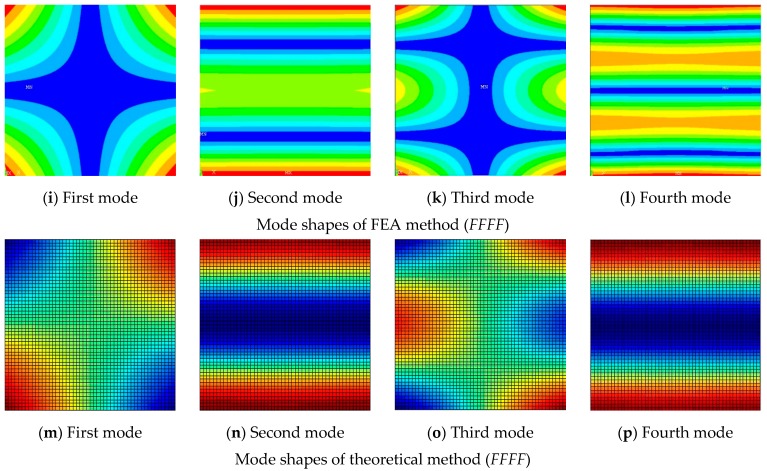
Mode shapes of the composite laminated plate. (**a**,**e**,**i**,**m**) First mode, (**b**,**f**,**j**,**n**) Second mode, (**c**,**g**,**k**,**o**) Third mode, (**d**,**h**,**l**,**p**) Fourth mode.

Figure 3 shows the mode shapes of the *CCCC* and *FFFF* moderately thick laminated composite plate. The mode shapes obtained by the present method coincide with those obtained by the finite element method. The material properties were as follows: a/b = 1, h/b = 0.2; the layup direction of the unit plate was 0°/90°/0°.

**Figure 4:**
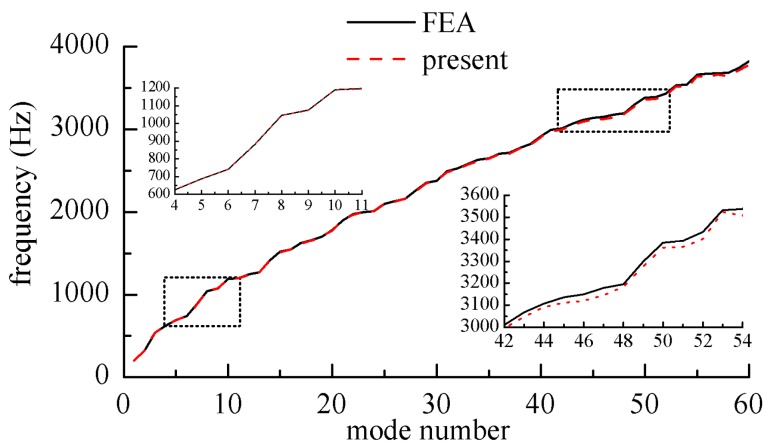
Nature frequency parameters for rectangular laminated plates with *CFSF*.

**Figure 5:**
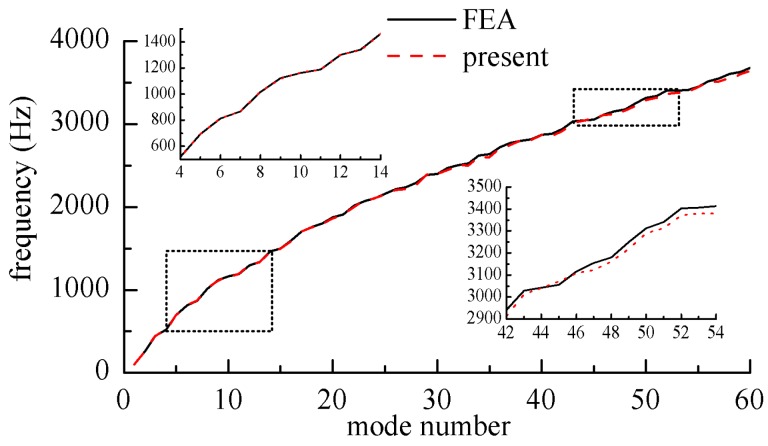
Nature frequency parameters for rectangular laminated plates with *ECFF*.

In order to show the consistency between the results of the present method and finite element method clearly, Figure 4 shows the frequency parameter curves of the *SFCF* laminated composite plates. The layup direction of the unit plate was 45°/−45°/45°; *a*/*b* = 1; *h*/*b* = 0.1. Figure 5 shows the frequency parameter curves of the *EFCS* laminated composite plates. The spring stiffness coefficient on the elastic edge were as follows: *k*_x0_ = *D*_0_, *K*_x0_ = 0, *K*_yx0_ = 0.

In Figure 4 and Figure 5, the frequency curves obtained by this method coincided with those obtained by the finite element method at medium and low frequencies. At high frequencies, the results obtained by the finite element method were larger than presented results. This was because the finite element method needs finer meshes in order to get more accurate results in the high frequency band, with the workload increasing sharply.

**Figure 6:**
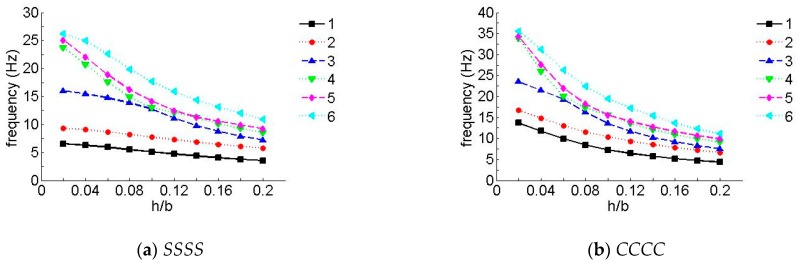
Natural frequency parameters for a laminate with an arbitrary ratio between thickness and width (three layers). (**a**) *SSSS*, (**b**) *CCCC*.

**Figure 7:**
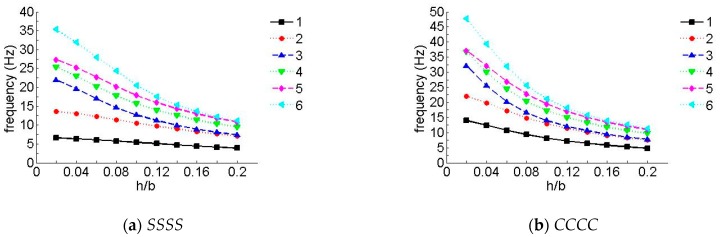
Natural frequency parameters for a laminate with an arbitrary ratio between thickness and width (five layers). (**a**) *SSSS*, (**b**) *CCCC*.

To research the influence of different ratios between thickness and width, the first six non-dimensional frequency parameters for the *SSSS* and *CCCC* laminated composite plates are shown in Figure 6 and Figure 7, where the number of piles in the figures were three layers and five layers, respectively. As can be seen in the figures, compared with low-order frequency parameters, high-order frequency parameters showed a remarkable decrease with an increase in the ratio between thickness and width; moreover, the change was more significant when *h/b* was less than 0.10.

**Figure 8:**
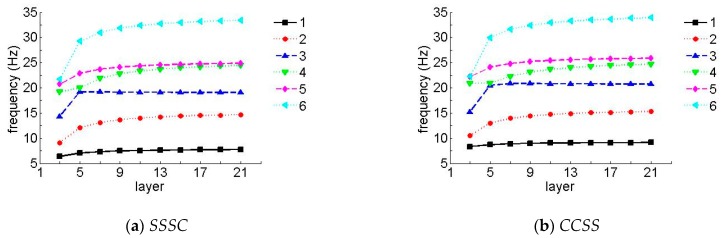
Natural frequency parameters for a laminate with arbitrary layers (laying angle = 45°). (**a**) *SSSC*, (**b**) *CCSS*.

**Figure 9:**
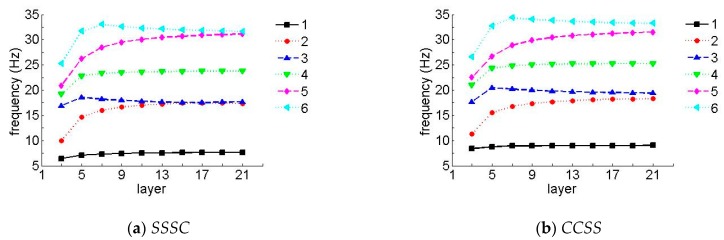
Natural frequency parameters for a laminate with arbitrary layers (laying angle = 90°). (**a**) *SSSC*, (**b**) *CCSS*.

To further verify the effect of the number of layers on the frequency, Figure 8 and Figure 9 show the first six non-dimensional frequency parameters for the *SSSC* and *CCSS* laminated composite plates of various layers, where *h/b* = 0.05 and the layup directions of the unit plates in Figure 8 and Figure 9 were 0°/45°/0° and 0°/90°/0°, respectively (when the number of layers was three), and the layup directions in Figure 8 and Figure 9, respectively were 0°/45°/0°/45°/0° and 0°/90°/0°/90°/0° (when the number of layers is five), etc. In the following figures, the data were collected when the number of layers was odd (3, 5, 7, 9, etc.).

It can be seen in Figure 8 and Figure 9 that the frequency parameters increased with the increase in the number of layers, so long as the number of layers was less than 13, in which case, it stayed constant as the number of layers changed. It should be mentioned that the effect was more noticeable for higher frequencies.

**Figure 10:**
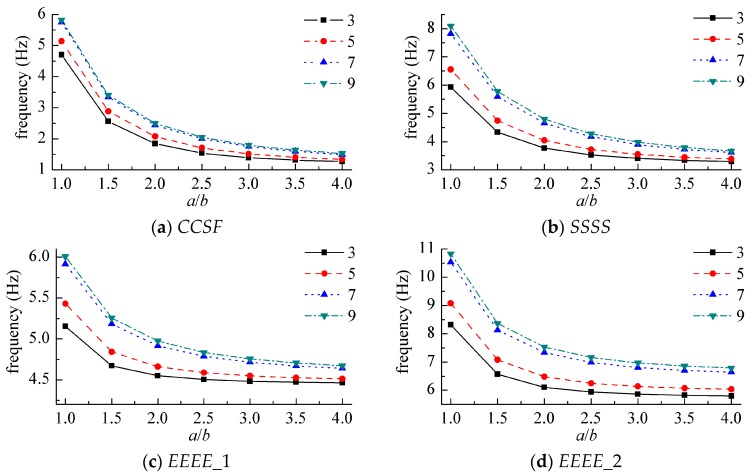
Natural frequency parameters for symmetric angle-ply laminates. (**a**) *CCSF*, (**b**) *SSSS*, (**c**) *EEEE*_1, (**d**) *EEEE*_2.

In order to study the influence of the number of layers laid on the vibration frequency, Figure 10 shows the variation of the dimensionless frequency of the first order with the number of layers and the aspect ratio of the normal symmetric angled plywood structure under different boundary conditions. Among them, the laying angle of the laminate was 45°, and *h/b* = 0.1.

In Figure 10c,d, the spring stiffness coefficients on each side of *EEEE*_1 were *k_x_*_0_ = *K_x_*_0_ = *K_yx_*_0_ = 10^6^ × *D*_0_, *k_y_*_0_ = *K_y_*_0_ = *K_xy_*_0_ = 10^4^ × *D*_0_, *k_xa_* = *K_xa_* = *K_yxa_* = 10^2^ × *D*_0_, *k_yb_* = *K_yb_* = *K_xyb_* = 10^3^ × *D*_0_; the spring stiffness coefficients of *EEEE*_2 were *k_x_*_0_ = *k_y_*_0_ = *k_xa_* = *k_yb_* = 10^6^ × *D*_0_, *K_x_*_0_ *= K_y_*_0_ = *K_xa_* = *K_yb_* = 10^4^ × *D*_0_, *K_yx_*_0_ = *K_xy_*_0_ = *K_yxa_* = *K_xyb_* = 10^2^ × *D*_0_.

**Figure 11:**
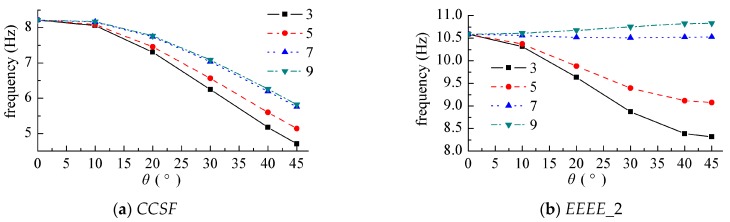
Natural frequency parameters for symmetric angle-ply laminates. (**a**) *CCSF*, (**b**) *EEEE*_2.

The laying angle was another important design parameter of the laminate. Figure 11 shows the variation of the first-order vibration frequency with the laying angle of the composite laminate under different layers.

### 3.2. Vibration Response Analysis of Laminated Composite Plates

In this section, the following example is focused on the vibration harmonic response of the plate. In order to avoid the numerical instability under the excitation of the plate structure resonance frequency in the numerical calculation, the structural damping factor η is introduced in the analysis, and the elastic modulus of the plate structure is correspondingly the complex elastic modulus, *E^’^* = *E*(1 + *jη*). To avoid the instability of the response at the resonant frequency, the damping factor η (loss factor value) of the plate structure herein is η = 0.01.

The material in this section is an orthotropic plate, and the material properties of the plate were as follows: a = 1 m, b = 1 m, h = 0.1 m, *E_1_* = 128.8 GPa, *E_2_* = 8.3 GPa, *G_12_* = 4.1 GPa, *G_23_* = *G_12_*, *G_13_* = *G_12_*, *v_2_* = 0.355.

**Figure 12:**
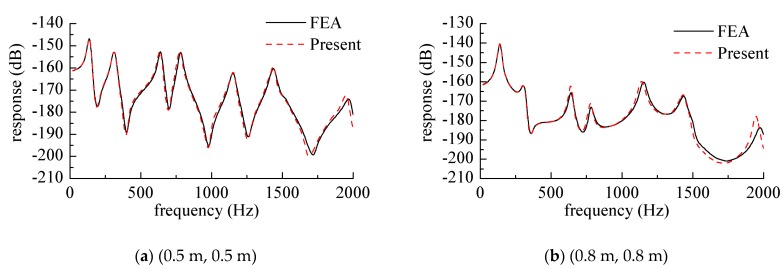
Response curve while the harmonic force acted on the center of the cantilever plate. (**a**) (0.5 m, 0.5 m), (**b**) (0.8 m, 0.8 m).

Figure 12 shows the displacement harmonic response curve at different points when the point force acted on the center of the cantilever plate. This part of the response curve was based on the frequency domain analysis, as the comparison map obtained by the frequency domain analysis was more intuitive, and the full text always analyzed the frequency domain part of the research, which was also to maintain the consistency of the whole article.

The solid support edge was the edge on *x* = 0, and the force amplitude was *F* = 1 N. Figure 12a is a curve where the response point was at the center of the plate (0.5 m, 0.5 m), and Figure 12b is a curve at which the response point was at (0.8 m, 0.8 m). It can also be seen from the figure that the frequency corresponding to the peak value of the response was the modal frequency of the plate structure, and the response curves obtained by the finite element method were also given in the two figures. The mesh size was 0.02 m × 0.02 m.

**Figure 13:**
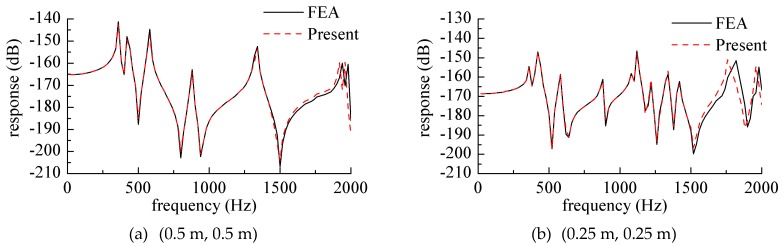
Response curve while the harmonic force acted on various positions of the cantilever plate. (**a**) (0.5 m, 0.5 m), (**b**) (0.25 m, 0.25 m).

In order to study the effect of the excitation on the response of the rectangular plates with different positions, Figure 13 shows the displacement response and velocity response of the composite laminate under the action of point force. The boundary condition of the laminate was *CFFC*. The amplitude of the point force was 1 N, the action direction was along the *z*-axis, the action position was (0.5 m, 0.5 m) and the action time was 0.5 s. The displacement response in the figure was the same as the position of the velocity response, with both points being (0.5 m, 0.5 m). The calculation curve obtained by the finite element method is also shown in the figure. It can be seen that the results obtained by the two methods agreed well, thus verifying the feasibility of the method for solving transient problems.

### 3.3. Vibration Power Flow Analysis of Laminated Composite Plates

In some engineering applications, studying the energy transfer path in structural systems is helpful for solving the problems of structural vibration and noise radiation. In recent years, as a new energy transfer analysis method, power flow studies the transmission and control of vibration in structures from the perspective of energy. Because the power flow takes into account the inherent information of both force and motion of the structure, it can evaluate the vibration response characteristics of the structure more comprehensively than the previous single description of force or displacement. In addition, energy dissipation and collection can be characterized through the study of power flow fields, having practical significance for engineering.

**Figure 14:**
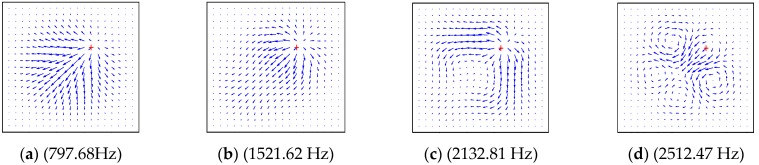
The power flow field for a *CCCC* laminated plate excited by point force on (0.65 m, 0.65 m). (**a**) (797.68Hz), (**b**) (1521.62 Hz), (**c**) (2132.81 Hz), (**d**) (2512.47 Hz).

**Figure 15:**
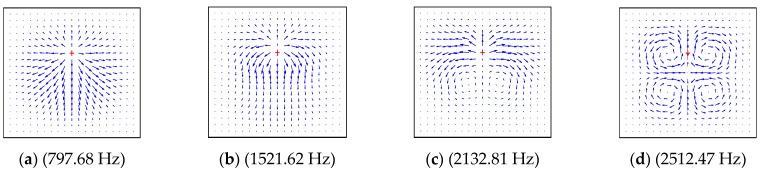
The power flow field for a *CCCC* laminated plate excited by point force on (0.5 m, 0.65 m). (**a**) (797.68Hz), (**b**) (1521.62 Hz), (**c**) (2132.81 Hz), (**d**) (2512.47 Hz).

**Figure 16:**
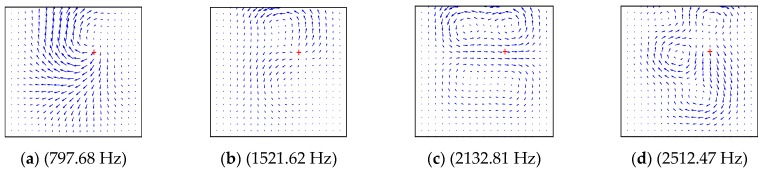
The power flow field for a *CSSE* laminated plate excited by point force on (0.65 m, 0.65 m). (**a**) (797.68Hz), (**b**) (1521.62 Hz), (**c**) (2132.81 Hz), (**d**) (2512.47 Hz).

**Figure 17:**
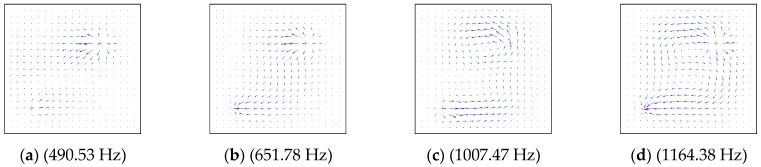
The power flow field for a *CSSS* lamitated plate excited by point force on (0.2 m, 0.2 m) and (0.7 m, 0.7 m). (**a**) (490.53 Hz), (**b**) (651.78 Hz), (**c**) (1007.47 Hz), (**d**) (1164.38 Hz).

In order to study the influence factors and characteristics of energy transfer, the dynamical characteristics of the structures are described as the power flow field. The power flow intensity at any point can be obtained by extracting the force and displacement from Equations (37)–(39), as shown in Figure 14, Figure 15, Figure 16 and Figure 17 by using MATLAB 2017 this section, the effects of boundary conditions, the excitation frequency and the excitation location on energy transfer characteristics are presented.

The material in this part was normal symmetric orthogonally laid laminate, and the material properties of the plate were as follows: *a* = 1 m, *b* = 1 m, *h* = 0.1 m, *E*_1_ = 128.8 GPa, *E*_2_ = 8.3 GPa, *G*_12_ = 4.1 GPa, *G*_23_ = *G*_12_, *G*_13_ = *G*_12_, *v*_2_ = 0.355. The boundary condition of Figure 14 and Figure 15 was *CCCC*; the four subgraphs given in each figure show the power flow field under different excitation frequencies, which were the first four natural frequencies of the composite plate. By comparing the four subgraphs, it can be seen that the distribution field and frequency of the energy flow had a significant influence. When the excitation force, action position and boundary conditions remain unchanged, the frequency change may also cause a change in the power flow distribution field. 

Figure 16 shows the power flow distribution of the plate structure under different excitation frequencies when the boundary condition was *CSSE* and the excitation force acted on the point (0.65 m, 0.65 m). The stiffness coefficients of the three types of elastic on the elastic side were *k_y_*_b_ = *K_xyb_* = *D*_0_, *K_yb_* = 0.

The excitation frequency in Figure 16 was not the first four natural frequencies of the *CSSE* rectangular plate, but the excitation frequency from Figure 14. It can be seen from the figure that the distribution of the power flow field had strong dependence on the position of the excitation force and the boundary condition of the rectangular plate, but there was no fixed distribution between them.

In order to study the energy transfer characteristics of plate structures subjected to multiple point forces, Figure 17 shows the distribution of vibration power flow in the whole rectangular plate structure under two-point excitation. The boundary conditions of the plate structure were *CSSS*. The excitation frequencies of the two point forces were the same, and the direction of action was along the *z*-axis, with the amplitude of 1 N. The positions of action for the two points were (0.2 m, 0.2 m) and (0.7 m, 0.7 m), respectively.

Figure 17a–d shows the power flow vector graphs at first to fourth natural frequencies. It can be seen from the figure that the vibration energy was transmitted from the excitation to the surrounding plate, but the excitation position was not always the source of vibration energy output. Under some excitation frequencies, the excitation position may also have the flow of vibration energy, which further verifies that the energy transfer was strongly dependent on the excitation frequency.

In Figure 14, Figure 15, Figure 16 and Figure 17, it can be seen that the energy transfer in the structure had an important relationship with the frequency, position, number of excitation forces and boundary conditions of the plate structure. As the excitation frequency increased, the flow path of the vibration energy in the structure became more and more complicated. When the boundary condition of one side became soft, the vibration energy rapidly flowed to this side. The vibration energy did not always transfer energy from the excitation position to the boundary according to the shortest path principle. There was also a certain form of energy circulation inside the plate structure. Through the description of the power flow, the transmission of the vibration energy could be clearly seen, thereby providing a basis for the effective control of the vibration of the structure.

### 3.4. Modal Test of Laminated Plates

**Figure 18:**
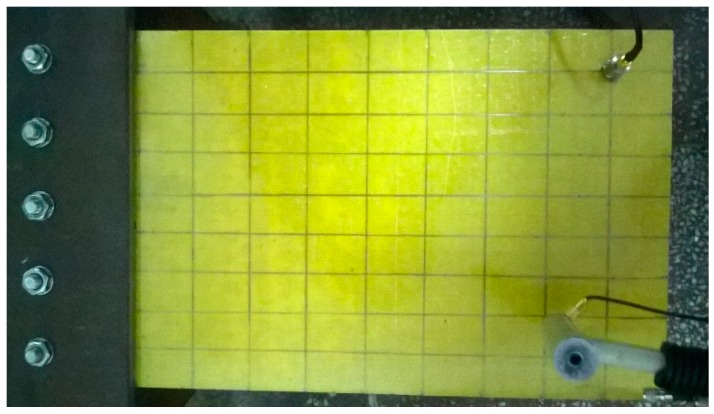
The test model of the laminated plate.

Aiming at strengthening our understanding of the vibration behaviors of the laminated composite plate, we verified the accuracy of the present method by experimental means. Figure 18 shows the experiment to study the model characteristics of the plate. A hammer was used for excitation, and the experiment was carried out by single-point pock-up and point-by-point excitation.

The composite laminate used in the experiment consisted of four layers of anisotropic single-layer plates. The material of the single-layer plate was epoxy-based composite, and the material properties of the plate were as follows: *a* = 0.29116 m, *b* = 0.19934 m, *h* = 0.00502 m, *E*_1_ = 135 GPa, *E*_2_ = 8.3 GPa, *G*_12_ = 4.1 GPa, *G*_23_ = *G*_12_, *G*_13_ = *G*_12_, *v*_2_ = 0.355.

**Figure 19:**
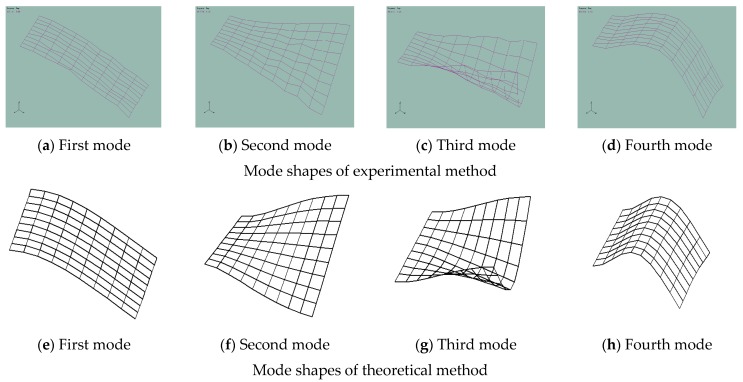
Comparisons of modal shapes of the laminated plate between measured and theoretical results. (**a**) First mode, (**b**) Second mode, (**c**) Third mode, (**d**) Fourth mode, (**e**) First mode, (**f**) Second mode, (**g**) Third mode, (**h**) Fourth mode.

In this experiment, the boundary condition of the laminated composite plate was *CFFF*, and the clamped boundary condition was simulated by the pressure plate and the *Φ6* bolt fixed on the foundation. The laminated plate was divided into a uniform mesh, the number of grids was 9 × 9 and the excitation position of the hammer was at each grid point. According to the experimental requirements, the sampling frequency was selected to be 5000 Hz and the analysis frequency was 1950 Hz, where the sampling mode was transient and the triggering mode was signal triggering. The Donghua DH5922 data acquisition analyzer and dynamic signal acquisition system were used to analyze the excitation signal of the hammer and the response signal of the acceleration sensor. Finally, a modal analysis was performed to obtain the natural frequency in Table 6 and the mode shape of the rectangular plate in Figure 19.

Table 6 shows the first eight natural frequency values obtained from the experimental composite laminate structure. The theoretical results are also given in the table. By comparing the experimental values and the theoretical values, we see that the results were in good agreement, and the maximum deviation was less than 4%, which verified the accuracy of the proposed modeling method. The results of the two methods showed a certain deviation, mainly because during the experiment the clamped boundary conditions were simulated by using multiple bolt joints, which had certain inconsistencies with the complete solidification in the theoretical calculation process. This may cause the stiffness to be less than the clamped end stiffness, resulting in the frequency of the experiment being higher than the theoretical value. Furthermore, a certain error was also generated, as the acceleration sensor introduced additional mass when it was connected to the plate structure.

Figure 19 shows the first four natural modes of the composite laminate obtained by the experimental method and theoretical method.

## 4. Summary

In this paper, on the basis of the improved Fourier series method, vibration modeling of a laminated composite plate was proposed to study vibration characteristics of laminates. According to the Mindlin plate theory, the mechanical models of the laminates were established and a number of functions were deduced, such as the potential energy function, as well as the function of kinetic energy. Simultaneously, according to the Hamilton principle, the vibration equations of the plates with arbitrary boundary conditions were derived by expressing the displacement as a superposition of a Fourier cosine series and four auxiliary polynomials.

Through the numerical analysis, the effectiveness of the present method was verified by a comparison between the FEA results and the results of the method proposed in relevant references and mode shapes. The natural frequency was analyzed in terms of the different boundary conditions, and the present method had the better accuracy. The effects of boundary conditions, the ratio between thickness and width, the number of layers of laminates and the laying angle on the vibration characteristics of composite laminates were studied. 

It can be seen that the vibration frequency of laminate increased as the spring stiffness on the boundary increased. As the number of layers increased, the frequencies, except for the first-order frequency, showed a noticeable increase, and the frequency eventually became stable when the number of layers increased to a certain value. It can also be concluded that the effect of frequency regarding the ratio between thickness and width is significant; with the increase in h/b, the frequency of the plate vibration decreased. 

Furthermore, in order to study the influencing factors and characteristics of the energy transfer, the harmonic response analysis and power flow analysis of the composited plate vibration showed that the vibration energy does not always transfer energy to the boundary according to the shortest path principle, and that there is also a certain form of energy cycle inside the plate structure. The distribution field and frequency of the energy flow had a significant influence. When the excitation force, excitation position and boundary conditions remain unchanged, the frequency change may also cause a complete change in the energy transfer.

## Figures and Tables

**Table 1:** Spring stiffness under different boundary conditions.

BC	*k_x_*_0_	*K_x_*_0_	*K_yx_*_0_
*F*	0	0	0
*S*	$10^{6}\times D_{0}$	0	$10^{6}\times D_{0}$
*C*	$10^{6}\times D_{0}$	$10^{6}\times D_{0}$	$10^{6}\times D_{0}$
*E*	(0,∞)	(0,∞)	(0,∞)

**Table 2:** Natural frequency parameters for laminated plates with different methods.

BC	Modes	h/b = 0.1	Ref. [18]	Error	h/b = 0.2	Ref. [18]	Error
		Present			Present		
*CCCC*	*f*_1_	3.2954	3.2954	0.000%	2.6876	2.6874	0.007%
	*f*_2_	6.2860	6.2858	0.003%	4.6909	4.6907	0.004%
	*f*_3_	6.2860	6.2858	0.003%	4.6909	4.6907	0.004%
	*f*_4_	8.8105	8.8099	0.007%	6.299	6.2984	0.010%
	*f*_5_	10.3787	10.3787	0.000%	7.1766	7.1766	0.000%
*CSSF*	*f*_1_	2.2368	2.2363	0.022%	1.8823	1.8821	0.011%
	*f*_2_	3.6012	3.601	0.006%	2.8901	2.89	0.003%
	*f*_3_	5.4904	5.4895	0.016%	4.1419	4.1417	0.005%
	*f*_4_	6.5979	6.5977	0.003%	4.9848	4.9843	0.010%
	*f*_5_	6.7808	6.7804	0.006%	5.0088	5.0086	0.004%
*ECCS*	*f*_1_	2.8975	2.8974	0.003%	2.3846	2.3843	0.013%
	*f*_2_	4.5886	4.5881	0.011%	3.6439	3.6434	0.014%
	*f*_3_	6.0365	6.0364	0.002%	4.4907	4.4902	0.011%
	*f*_4_	7.682	7.681	0.013%	5.5498	5.5488	0.018%
	*f*_5_	7.7098	7.7089	0.012%	5.7913	5.791	0.005%

**Table 3:** Convergence study of frequency for rectangular laminated plates with *SSFF*.

*M* = *N*	1	2	3	4	5	6	7
4	0.4325	2.4001	4.7328	5.4424	5.8221	7.6876	8.8219
5	0.4275	2.3972	4.7319	5.4376	5.8136	7.6752	8.8146
8	0.4267	2.3965	4.7317	5.4355	5.8126	7.6715	8.8140
10	0.4266	2.3964	4.7317	5.4350	5.8125	7.6706	8.8139
12	0.4266	2.3963	4.7317	5.4347	5.8124	7.6702	8.8139
14	0.4265	2.3963	4.7317	5.4346	5.8124	7.6700	8.8139
15	0.4265	2.3963	4.7317	5.4346	5.8124	7.6699	8.8139
16	0.4265	2.3963	4.7317	5.4345	5.8124	7.6699	8.8139
Ref. [21]	0.426	2.396	4.732	5.434	5.812	7.670	8.814

**Table 4:** Natural frequency parameters for rectangular laminated plates with *SSSS*, *CCCC*, *SCSC* and *CFFF*.

BC	Modes	h/b = 0.05			h/b = 0.1			h/b = 0.2		
		Present	Ref. [21]	FEA	Present	Ref. [21]	FEA	Present	Ref. [21]	FEA
*SSSS*	*f*_1_	6.134	6.138	6.149	5.164	5.166	5.188	3.593	3.594	3.608
	*f*_2_	8.884	8.888	8.881	7.755	7.757	7.737	5.768	5.769	5.747
	*f*_3_	15.105	15.11	15.055	12.911	12.915	12.838	7.395	7.397	7.363
	*f*_4_	19.319	19.354	19.333	13.038	13.049	13.086	8.686	8.688	8.691
	*f*_5_	20.631	20.665	20.73	14.366	14.376	14.377	9.144	9.145	9.107
*CCCC*	*f*_1_	10.933	10.953	10.938	7.405	7.411	7.412	4.446	4.447	4.446
	*f*_2_	14.009	14.028	14.011	10.387	10.393	10.390	6.64	6.642	6.641
	*f*_3_	20.366	20.388	20.372	13.897	13.913	13.895	7.698	7.7	7.696
	*f*_4_	23.111	23.196	23.115	15.419	15.429	15.419	9.183	9.185	9.185
	*f*_5_	24.897	24.978	24.903	15.79	15.806	15.802	9.735	9.738	9.735
*SCSC*	*f*_1_	6.887	6.89	6.893	5.868	5.871	5.893	4.136	4.137	4.157
	*f*_2_	11.241	11.246	11.208	9.45	9.454	9.483	6.473	6.474	6.438
	*f*_3_	18.651	18.664	18.641	13.33	13.34	13.367	7.663	7.664	7.692
	*f*_4_	19.585	19.619	19.593	14.869	14.878	14.883	9.157	9.159	9.168
	*f*_5_	21.768	21.801	21.74	15.329	15.34	15.491	9.641	9.643	9.657
*CFFF*	*f*_1_	2.126	2.127	2.129	1.918	1.918	1.925	1.444	1.444	1.436
	*f*_2_	2.37	2.369	2.386	2.103	2.103	2.093	1.545	1.545	1.541
	*f*_3_	4.562	4.559	4.536	4.188	4.188	4.162	3.466	3.466	3.472
	*f*_4_	10.045	10.04	10.049	7.752	7.757	7.749	4.686	4.687	4.655
	*f*_5_	11.056	11.07	11.063	7.957	7.961	7.943	4.86	4.86	4.877

**Table 5:** Natural frequency parameters for rectangular laminated plates with SCSF, SSSC, SSSF and SFSF.

BC	Modes	h/b = 0.05			h/b = 0.1			h/b = 0.2		
		Present	Ref. [18]	FEA	Present	Ref. [18]	FEA	Present	Ref. [18]	FEA
*SCSF*	*f*_1_	5.826	5.842	5.841	4.863	4.91	4.879	3.287	3.348	3.302
	*f*_2_	7.137	7.124	7.143	6.071	6.091	6.099	4.313	4.339	4.335
	*f*_3_	11.583	11.548	11.555	9.886	9.847	9.841	7.013	6.956	6.984
	*f*_4_	19.126	19.041	19.123	12.888	13.16	12.939	7.237	7.424	7.211
	*f*_5_	19.149	19.393	19.162	13.491	13.736	13.424	7.798	7.954	7.839
*SSSC*	*f*_1_	6.425	6.45	6.437	5.448	5.496	5.485	3.834	3.88	3.852
	*f*_2_	9.978	9.983	9.963	8.584	8.577	8.56	6.139	6.103	6.099
	*f*_3_	16.839	16.796	16.762	13.155	13.426	13.199	7.511	7.687	7.572
	*f*_4_	19.424	19.675	19.636	13.908	13.796	13.868	8.929	9.031	8.972
	*f*_5_	21.138	21.369	21.326	14.822	15.041	14.911	9.4	9.256	9.367
*SSSF*	*f*_1_	5.781	5.801	5.796	4.819	4.87	4.835	3.239	3.303	3.269
	*f*_2_	6.655	6.65	6.666	5.639	5.669	5.678	4.017	4.058	4.047
	*f*_3_	10.301	10.278	10.287	8.975	8.965	8.963	6.654	6.624	6.613
	*f*_4_	17.281	17.223	17.212	12.869	13.143	12.919	7.214	7.403	7.248
	*f*_5_	19.131	19.377	19.343	13.295	13.549	13.334	7.641	7.808	7.692
*SFSF*	*f*_1_	5.731	5.756	5.745	4.779	4.834	4.825	3.212	3.279	3.253
	*f*_2_	5.93	5.929	5.944	4.933	4.968	4.959	3.311	3.365	3.319
	*f*_3_	7.398	7.357	7.404	6.319	6.324	6.35	4.619	4.645	4.646
	*f*_4_	11.925	11.864	11.897	10.347	10.311	10.316	7.194	7.385	7.207
	*f*_5_	19.09	19.339	19.102	12.841	13.118	12.992	7.271	7.451	7.339

**Table 6:** Natural frequency of the laminate plate.

Mode	Experiment (Hz)	Present (Hz)	Error (%)
1	88.71	86.85	2.10
2	127.57	124.98	2.03
3	360.23	351.55	2.41
4	554.34	537.23	3.08
5	610.18	586.63	3.86
6	798.82	782.42	2.05
7	881.37	851.30	3.41
8	1245.52	1209.85	2.86

## Appendix A

The detailed expressions of the submatrices *K_ij_* and *M_ij_* in Equations (34)–(35) are provided as follows.

(A1) $${\left\{M_{1,1}\right\}}_{s,t}=\rho h\int_{0}^{a}\int_{0}^{b}\cos\lambda_{m1}x\cos\lambda_{m}x\cos\lambda_{n1}y\cos\lambda_{n}ydxdy$$

(A2) $${\left\{M_{1,2}\right\}}_{s,t}=\rho h\int_{0}^{a}\int_{0}^{b}\cos\lambda_{m1}x\cos\lambda_{m}x\xi_{1b}(y)\cos\lambda_{n}ydxdy$$

(A3) $${\left\{M_{1,3}\right\}}_{s,t}=\rho h\int_{0}^{a}\int_{0}^{b}\cos\lambda_{m1}x\cos\lambda_{m}x\xi_{2b}(y)\cos\lambda_{n}ydxdy$$

(A4) $${\left\{M_{1,4}\right\}}_{s,t}=\rho h\int_{0}^{a}\int_{0}^{b}\xi_{1a}(x)\cos\lambda_{m}x\cos\lambda_{n1}y\cos\lambda_{n}ydxdy$$

(A5) $${\left\{M_{1,5}\right\}}_{s,t}=\rho h\int_{0}^{a}\int_{0}^{b}\xi_{2a}(x)\cos\lambda_{m}x\cos\lambda_{n1}y\cos\lambda_{n}ydxdy$$

where *s* = *m*(*N* + 1) + *n* + 1, *t* = *m*1 (*N* + 1) + *n*1 + 1, *m* = 0, 1, …, *M*, *m*1 = 0, 1, …, *M*, *n* = 0, 1, …, *N*, *n*1 = 0, 1, …, *N*, *M* and *N* are the cutoff value. And the submatrix ***M***_1,6_ to ***M***_1,15_ are zero matrix. Similarily, the other submatrix can be expressed as

(A6) $${\left\{M_{2,1}\right\}}_{s,t}=\rho h\int_{0}^{a}\int_{0}^{b}\cos\lambda_{m1}x\cos\lambda_{m}x\xi_{1b}(y)\cos\lambda_{n1}ydxdy$$

(A7) $${\left\{M_{2,2}\right\}}_{s,t}=\rho h\int_{0}^{a}\int_{0}^{b}\cos\lambda_{m1}x\cos\lambda_{m}x\xi_{1b}(y)\xi_{1b}(y)dxdy$$

(A8) $${\left\{M_{2,3}\right\}}_{s,t}=\rho h\int_{0}^{a}\int_{0}^{b}\cos\lambda_{m1}x\cos\lambda_{m}x\xi_{1b}(y)\xi_{2b}(y)dxdy$$

(A9) $${\left\{M_{2,4}\right\}}_{s,t}=\rho h\int_{0}^{a}\int_{0}^{b}\xi_{1a}(x)\cos\lambda_{m}x\xi_{1b}(y)\cos\lambda_{n1}ydxdy$$

(A10) $${\left\{M_{2,5}\right\}}_{s,t}=\rho h\int_{0}^{a}\int_{0}^{b}\xi_{2a}(x)\cos\lambda_{m}x\xi_{1b}(y)\cos\lambda_{n1}ydxdy$$

(A11) $${\left\{M_{4,1}\right\}}_{s,t}=\rho h\int_{0}^{a}\int_{0}^{b}\cos\lambda_{m1}x\xi_{1a}(x)\cos\lambda_{n1}y\cos\lambda_{n}ydxdy$$

(A12) $${\left\{M_{4,2}\right\}}_{s,t}=\rho h\int_{0}^{a}\int_{0}^{b}\cos\lambda_{m1}x\xi_{1a}(x)\xi_{1b}(y)\cos\lambda_{n}ydxdy$$

(A13) $${\left\{M_{4,3}\right\}}_{s,t}=\rho h\int_{0}^{a}\int_{0}^{b}\cos\lambda_{m1}x\xi_{1a}(x)\xi_{2b}(y)\cos\lambda_{n}ydxdy$$

(A14) $${\left\{M_{4,4}\right\}}_{s,t}=\rho h\int_{0}^{a}\int_{0}^{b}\xi_{1a}(x)\xi_{1a}(x)\cos\lambda_{n1}y\cos\lambda_{n}ydxdy$$

(A15) $${\left\{M_{4,5}\right\}}_{s,t}=\rho h\int_{0}^{a}\int_{0}^{b}\xi_{1a}(x)\xi_{2a}(x)\cos\lambda_{n1}y\cos\lambda_{n}ydxdy$$

where the submatrix ***M***_2,6_ to ***M***_2,15_ and ***M***_4,6_ to ***M***_4,15_ are zero matrix.

(A16) $$\begin{array}{ll} {\left\{K_{1,1}\right\}}_{s,t} & =A_{55}\int_{0}^{a}\int_{0}^{b}\lambda_{m1}\lambda_{m}\sin\lambda_{m1}x\sin\lambda_{m}x\cos\lambda_{n1}y\cos\lambda_{n}ydxdy\\ & +A_{44}\int_{0}^{a}\int_{0}^{b}\lambda_{n1}\lambda_{n}\cos\lambda_{m1}x\cos\lambda_{m}x\sin\lambda_{n1}y\sin\lambda_{n}ydxdy\\ & +A_{45}\int_{0}^{a}\int_{0}^{b}\lambda_{m1}\lambda_{n}\sin\lambda_{m1}x\cos\lambda_{m}x\cos\lambda_{n1}y\sin\lambda_{n}ydxdy\\ & +A_{45}\int_{0}^{a}\int_{0}^{b}\lambda_{m}\lambda_{n1}\cos\lambda_{m1}x\sin\lambda_{m}x\sin\lambda_{n1}y\cos\lambda_{n}ydxdy\\ & +[k_{y0}+{\left(-1\right)}^{n1+n}k_{yb}]\int_{0}^{a}\cos\lambda_{m1}x\cos\lambda_{m}xdx\\ & +[k_{x0}+{\left(-1\right)}^{m1+m}k_{xa}]\int_{0}^{b}\cos\lambda_{n1}y\cos\lambda_{n}ydy \end{array}$$

(A17) $$\begin{array}{ll} {\left\{K_{1,2}\right\}}_{s,t} & =A_{55}\int_{0}^{a}\int_{0}^{b}\lambda_{m1}\lambda_{m}\sin\lambda_{m1}x\sin\lambda_{m}x\cos\lambda_{n1}y\xi_{1b}(y)dxdy\\ & -A_{44}\int_{0}^{a}\int_{0}^{b}\lambda_{n1}\cos\lambda_{m1}x\cos\lambda_{m}x\sin\lambda_{n1}y\xi_{1b}^{'}(y)dxdy\\ & -A_{45}\int_{0}^{a}\int_{0}^{b}\lambda_{m1}\sin\lambda_{m1}x\cos\lambda_{m}x\cos\lambda_{n1}y\xi_{1b}^{'}(y)dxdy\\ & +A_{45}\int_{0}^{a}\int_{0}^{b}\lambda_{m}\lambda_{n1}\cos\lambda_{m1}\sin\lambda_{m}x\sin\lambda_{n1}y\xi_{1b}(y)dxdy\\ & +[k_{x0}+{\left(-1\right)}^{m1+m}k_{xa}]\int_{0}^{b}\cos\lambda_{n1}y\xi_{1b}(y)dy \end{array}$$

(A18) $$\begin{array}{ll} {\left\{K_{1,3}\right\}}_{s,t} & =A_{55}\int_{0}^{a}\int_{0}^{b}\lambda_{m1}\lambda_{m}\sin\lambda_{m1}x\sin\lambda_{m}x\cos\lambda_{n1}y\xi_{2b}(y)dxdy\\ & -A_{44}\int_{0}^{a}\int_{0}^{b}\lambda_{n1}\cos\lambda_{m1}x\cos\lambda_{m}x\sin\lambda_{n1}y\xi_{2b}^{'}(y)dxdy\\ & -A_{45}\int_{0}^{a}\int_{0}^{b}\lambda_{m1}\sin\lambda_{m1}x\cos\lambda_{m}x\cos\lambda_{n1}y\xi_{2b}^{'}(y)dxdy\\ & +A_{45}\int_{0}^{a}\int_{0}^{b}\lambda_{m}\lambda_{n1}\cos\lambda_{m1}\sin\lambda_{m}x\sin\lambda_{n1}y\xi_{2b}(y)dxdy\\ & +[k_{x0}+{\left(-1\right)}^{m1+m}k_{xa}]\int_{0}^{b}\cos\lambda_{n1}y\xi_{2b}(y)dy \end{array}$$

(A19) $$\begin{array}{ll} {\left\{K_{1,4}\right\}}_{s,t} & =-A_{55}\int_{0}^{a}\int_{0}^{b}\lambda_{m1}\lambda_{m}\sin\lambda_{m1}x\xi_{1a}^{'}(x)\cos\lambda_{n1}y\cos\lambda_{n}ydxdy\\ & +A_{44}\int_{0}^{a}\int_{0}^{b}\lambda_{n1}\lambda_{n}\cos\lambda_{m1}x\xi_{1a}(x)\sin\lambda_{n1}y\sin\lambda_{n}ydxdy\\ & +A_{45}\int_{0}^{a}\int_{0}^{b}\lambda_{m1}\lambda_{n}\sin\lambda_{m1}x\xi_{1a}(x)\cos\lambda_{n1}y\sin\lambda_{n}ydxdy\\ & -A_{45}\int_{0}^{a}\int_{0}^{b}\lambda_{m}\lambda_{n1}\cos\lambda_{m1}x\xi_{1a}^{'}(x)\sin\lambda_{n1}y\cos\lambda_{n}ydxdy\\ & +[k_{y0}+{\left(-1\right)}^{n1+n}k_{yb}]\int_{0}^{a}\cos\lambda_{m1}x\cos\lambda_{m}xdx\\ & +[k_{x0}+{\left(-1\right)}^{m1+m}k_{xa}]\int_{0}^{b}\cos\lambda_{n1}y\cos\lambda_{n}ydy \end{array}$$

(A20) $$\begin{array}{ll} {\left\{K_{1,5}\right\}}_{s,t} & =-A_{55}\int_{0}^{a}\int_{0}^{b}\lambda_{m1}\lambda_{m}\sin\lambda_{m1}x\xi_{2a}^{'}(x)\cos\lambda_{n1}y\cos\lambda_{n}ydxdy\\ & +A_{44}\int_{0}^{a}\int_{0}^{b}\lambda_{n1}\lambda_{n}\cos\lambda_{m1}x\xi_{2a}(x)\sin\lambda_{n1}y\sin\lambda_{n}ydxdy\\ & +A_{45}\int_{0}^{a}\int_{0}^{b}\lambda_{m1}\lambda_{n}\sin\lambda_{m1}x\xi_{2a}(x)\cos\lambda_{n1}y\sin\lambda_{n}ydxdy\\ & -A_{45}\int_{0}^{a}\int_{0}^{b}\lambda_{m}\lambda_{n1}\cos\lambda_{m1}x\xi_{2a}^{'}(x)\sin\lambda_{n1}y\cos\lambda_{n}ydxdy\\ & +[k_{y0}+{\left(-1\right)}^{n1+n}k_{yb}]\int_{0}^{a}\cos\lambda_{m1}x\cos\lambda_{m}xdx\\ & +[k_{x0}+{\left(-1\right)}^{m1+m}k_{xa}]\int_{0}^{b}\cos\lambda_{n1}y\cos\lambda_{n}ydy \end{array}$$

(A21) $$\begin{array}{ll} {\left\{K_{1,6}\right\}}_{s,t}= & -A_{55}\int_{0}^{a}\int_{0}^{b}\lambda_{m1}\sin\lambda_{m1}x\cos\lambda_{m}x\cos\lambda_{n1}y\cos\lambda_{n}ydxdy\\ & -A_{45}\int_{0}^{a}\int_{0}^{b}\lambda_{n1}\cos\lambda_{m1}x\cos\lambda_{m}x\sin\lambda_{n1}y\cos\lambda_{n}ydxdy \end{array}$$

(A22) $$\begin{array}{ll} {\left\{K_{1,7}\right\}}_{s,t}= & -A_{55}\int_{0}^{a}\int_{0}^{b}\lambda_{m1}\sin\lambda_{m1}x\cos\lambda_{m}x\cos\lambda_{n1}y\xi_{1b}(y)dxdy\\ & -A_{45}\int_{0}^{a}\int_{0}^{b}\lambda_{n1}\cos\lambda_{m1}x\cos\lambda_{m}x\sin\lambda_{n1}y\xi_{1b}(y)dxdy \end{array}$$

(A23) $$\begin{array}{ll} {\left\{K_{1,8}\right\}}_{s,t}= & -A_{55}\int_{0}^{a}\int_{0}^{b}\lambda_{m1}\sin\lambda_{m1}x\cos\lambda_{m}x\cos\lambda_{n1}y\xi_{2b}(y)dxdy\\ & -A_{45}\int_{0}^{a}\int_{0}^{b}\lambda_{n1}\cos\lambda_{m1}x\cos\lambda_{m}x\sin\lambda_{n1}y\xi_{2b}(y)dxdy \end{array}$$

(A24) $$\begin{array}{ll} {\left\{K_{1,9}\right\}}_{s,t}= & -A_{55}\int_{0}^{a}\int_{0}^{b}\lambda_{m1}\sin\lambda_{m1}x\xi_{1a}(x)\cos\lambda_{n1}y\cos\lambda_{n}ydxdy\\ & -A_{45}\int_{0}^{a}\int_{0}^{b}\lambda_{n1}\cos\lambda_{m1}x\xi_{1a}(x)\sin\lambda_{n1}y\cos\lambda_{n}ydxdy \end{array}$$

(A25) $$\begin{array}{ll} {\left\{K_{1,10}\right\}}_{s,t}= & -A_{55}\int_{0}^{a}\int_{0}^{b}\lambda_{m1}\sin\lambda_{m1}x\xi_{2a}(x)\cos\lambda_{n1}y\cos\lambda_{n}ydxdy\\ & -A_{45}\int_{0}^{a}\int_{0}^{b}\lambda_{n1}\cos\lambda_{m1}x\xi_{2a}(x)\sin\lambda_{n1}y\cos\lambda_{n}ydxdy \end{array}$$

(A26) $$\begin{array}{ll} {\left\{K_{1,11}\right\}}_{s,t}= & -A_{44}\int_{0}^{a}\int_{0}^{b}\lambda_{n1}\cos\lambda_{m1}x\cos\lambda_{m}x\sin\lambda_{n1}ycos\lambda_{n}ydxdy\\ & -A_{45}\int_{0}^{a}\int_{0}^{b}\lambda_{m1}\sin\lambda_{m1}x\cos\lambda_{m}x\cos\lambda_{n1}y\cos\lambda_{n}ydxdy \end{array}$$

(A27) $$\begin{array}{ll} {\left\{K_{1,12}\right\}}_{s,t}= & -A_{44}\int_{0}^{a}\int_{0}^{b}\lambda_{n1}\cos\lambda_{m1}x\cos\lambda_{m}x\sin\lambda_{n1}y\xi_{1b}(y)dxdy\\ & -A_{45}\int_{0}^{a}\int_{0}^{b}\lambda_{m1}\sin\lambda_{m1}x\cos\lambda_{m}x\cos\lambda_{n1}y\xi_{1b}(y)dxdy \end{array}$$

(A28) $$\begin{array}{ll} {\left\{K_{1,13}\right\}}_{s,t}= & -A_{44}\int_{0}^{a}\int_{0}^{b}\lambda_{n1}\cos\lambda_{m1}x\cos\lambda_{m}x\sin\lambda_{n1}y\xi_{2b}(y)dxdy\\ & -A_{45}\int_{0}^{a}\int_{0}^{b}\lambda_{m1}\sin\lambda_{m1}x\cos\lambda_{m}x\cos\lambda_{n1}y\xi_{2b}(y)dxdy \end{array}$$

(A29) $$\begin{array}{ll} {\left\{K_{1,14}\right\}}_{s,t}= & -A_{44}\int_{0}^{a}\int_{0}^{b}\lambda_{n1}\cos\lambda_{m1}x\xi_{1a}(x)\sin\lambda_{n1}ycos\lambda_{n}ydxdy\\ & -A_{45}\int_{0}^{a}\int_{0}^{b}\lambda_{m1}\sin\lambda_{m1}x\xi_{1a}(x)\cos\lambda_{n1}y\cos\lambda_{n}ydxdy \end{array}$$

(A30) $$\begin{array}{ll} {\left\{K_{1,15}\right\}}_{s,t}= & -A_{44}\int_{0}^{a}\int_{0}^{b}\lambda_{n1}\cos\lambda_{m1}x\xi_{2a}(x)\sin\lambda_{n1}ycos\lambda_{n}ydxdy\\ & -A_{45}\int_{0}^{a}\int_{0}^{b}\lambda_{m1}\sin\lambda_{m1}x\xi_{2a}(x)\cos\lambda_{n1}y\cos\lambda_{n}ydxdy \end{array}$$
